# Association of rs11780592 Polymorphism in the Human Soluble Epoxide Hydrolase Gene (EPHX2) with Oxidized LDL and Mortality in Patients with Diabetic Chronic Kidney Disease

**DOI:** 10.1155/2021/8817502

**Published:** 2021-05-06

**Authors:** Stefanos Roumeliotis, Athanasios Roumeliotis, Aikaterini Stamou, Stylianos Panagoutsos, Vangelis G. Manolopoulos, Fotis Tsetsos, Marianthi Georgitsi, Vassilios Liakopoulos

**Affiliations:** ^1^Division of Nephrology and Hypertension, 1st Department of Internal Medicine, AHEPA Hospital, School of Medicine, Aristotle University of Thessaloniki, 54636 Thessaloniki, Greece; ^2^Department of Microbiology, AHEPA Hospital, School of Medicine, Aristotle University of Thessaloniki, 54636 Thessaloniki, Greece; ^3^Department of Nephrology, School of Medicine, Democritus University of Thrace, 68100 Alexandroupolis, Greece; ^4^Laboratory of Pharmacology, School of Medicine, Democritus University of Thrace, 68100 Alexandroupolis, Greece; ^5^Department of Molecular Biology and Genetics, Democritus University of Thrace, 68100 Alexandroupolis, Greece; ^6^1st Laboratory of Medical Biology-Genetics, School of Medicine, Aristotle University of Thessaloniki, 54124 Thessaloniki, Greece

## Abstract

Soluble epoxide hydrolase 2 (EPHX2) is an enzyme promoting increased cellular apoptosis through induction of oxidative stress (OS) and inflammation. The *EPHX2* gene which encodes soluble EPHX2 might be implicated in the pathogenesis and development of OS and atherosclerosis. We aimed to assess the possible association between two functional polymorphisms of the *EPHX2* gene (rs2741335 and rs11780592) with oxidized LDL (ox-LDL), carotid atherosclerosis, mortality, and cardiovascular (CV) disease in 118 patients with diabetic chronic kidney disease (CKD). At baseline, ox-LDL and carotid intima-media thickness (cIMT) were evaluated and all patients were followed for seven years with outcomes all-cause mortality and CV events. rs11780592 *EPHX2* polymorphism was associated with ox-LDL, cIMT, albuminuria, and hypertension. Compared to AG and GG, AA homozygotes had higher values of albuminuria, ox-LDL, and cIMT (*p* = 0.046, *p* = 0.003, and *p* = 0.038, respectively). These associations remained significant, even after grouping for the G allele. After the follow-up period, 42/118 patients died (30/60 with AA genotype, 11/42 with AG genotype, and 1/12 with GG genotype) and 49/118 experienced a new CV event (fatal or nonfatal). The Kaplan-Meier analysis revealed that patients with the AA genotype exhibited a significantly higher mortality risk, compared to patients with AG and GG genotypes (*p* = 0.006). This association became even stronger, when AG and GG genotypes were grouped (AA vs. AG/GG, *p* = 0.002). AA homozygotes were strongly associated with all-cause mortality in both univariate (hazard ratio (HR) = 2.74, confidence interval (CI) = 1.40–5.35, *p* = 0.003) and multivariate Cox regression analysis (HR = 2.61, CI = 1.32–5.17, *p* = 0.006). In conclusion, our study demonstrated that genetic variations of *EPHX2* gene were associated with increased circulating ox-LDL, increased cIMT, and all-cause mortality in diabetic CKD. Since *EPHX2* regulates the cholesterol efflux and the oxidation of LDL in foam cells and macrophages, our study suggests that a genetic basis to endothelial dysfunction and OS might be present in diabetic CKD.

## 1. Introduction

Chronic kidney disease (CKD) and type 2 diabetes mellitus (T2DM) are conditions with growing incidence and prevalence worldwide, characterized by high cardiovascular (CV) morbidity and increased mortality [1]. Epidemiologic data suggest that compared to T2DM patients with normal kidney function and nondiabetic patients with CKD, the risk of mortality and CV disease is much higher in patients with the combination of these two (diabetes and CKD) [2, 3]. Furthermore, in end-stage renal disease (ESRD) patients undergoing maintenance hemodialysis (HD), T2DM contributes to a 1.6-fold increase in the overall mortality risk [4]. This heavy atherogenic and CV burden of diabetic CKD and ESRD cannot be solely explained by traditional risk factors. During the past decade, oxidative stress (OS), defined as the disruption of balance between prooxidant and antioxidant molecules in favor of the former, has emerged as a novel risk factor for the onset and development of atherosclerosis and CV disease in CKD patients. OS is present even at the early stages of CKD, progresses along with disease severity, and is further exacerbated by dialysis procedures [5–7]. Increased OS status triggers inflammation resulting in a direct alteration of lipids, proteins, carbohydrates, and DNA. It is now known that the oxidative modification of low-density cholesterol (LDL) is the first crucial step leading to endothelial dysfunction, the hallmark of atherosclerosis [8].

Soluble epoxide hydrolase 2 (EPHX2) is an enzyme involved in the metabolic breakdown of anti-inflammatory, antiatherogenic arachidonic acid-induced eicosanoids to proinflammatory diol molecules. This enzyme increases cellular apoptosis, through promotion of OS and inflammation. Since EPHX2 is encoded by the *EPHX2* gene, it has been hypothesized that genetic variations of this gene might alter the enzyme's function and activity. In vitro and in vivo studies suggest that EPHX2 might be implicated in the pathogenesis and development of OS, endothelial dysfunction, and atherosclerosis [9]. Moreover, epidemiologic data have repeatedly reported that genetic variations of the *EPHX2* gene are associated with the onset of CV disease in several populations [10, 11], and thus, *EPHX2* might be a potential atherosclerosis-susceptibility gene. Since inhibition of the activity of soluble EPHX2 has been reported to improve several aspects of CV disease, including OS, hypercholesterolemia, inflammation, hypertension, endothelial dysfunction, and atherosclerosis, EPHX2 is now regarded as an emerging therapeutic target in the treatment of atherosclerosis and CV disease [12, 13]. Although several studies have examined the association between various polymorphisms of the *EPHX2* gene and CV outcomes, there are no data regarding the role of rs2741335 and rs11780592 EPHX2 polymorphisms in atherosclerosis and endothelial dysfunction. Having this background in mind, the aim of our study was to investigate the possible association between rs2741335 and rs11780592 *EPHX2* polymorphisms with oxidized LDL (ox-LDL), carotid atherosclerosis, mortality, and CV disease in a cohort of T2DM patients with various degrees of renal function.

## 2. Materials and Methods

### 2.1. Patients

We recruited a total of 118 Caucasian, unrelated, adult patients (54 male, 64 female) with mean age 67.8 ± 8.7 years and with established T2DM for at least 6 years that were regularly followed in the Diabetic CKD Clinic of the University General Hospital of Alexandroupolis, Greece. At baseline (first visit), clinical and anthropometric data and background information regarding the history of CV disease were recorded, blood and urine samples were obtained, and carotid intima-media thickness (cIMT) was assessed. CV events were defined as documented stroke, myocardial infraction, coronary heart disease, angina, or peripheral artery disease. At enrolment, we measured albuminuria and calculated estimated glomerular filtration rate (eGFR) using the CKD epidemiology collaboration (CKD-EPI) equation [14] and every patient was classified in CKD stages, according to the Clinical Practice Guidelines for Chronic Kidney Disease established by the National Kidney Foundation's Kidney Disease Outcomes Quality Initiative [15]. Based on UACR and eGFR, at baseline, our study population included 17 patients in G1A1 stage, 2 in G1A2, 16 in G2A1, 9 in G2A2, 1 in G2A3, 3 in G3aA1, 10 in G3aA2, 2 in G3aA3, 3 in G3bA1, 11 in G3bA2, 4 in G3bA3, 3 in G4A1, 3 in G4A2, 5 in G4A3, and 29 in G5 stage (maintenance HD). Sixty-six patients received antiplatelets and 108 were under antihypertensive medication (65 used diuretics, 49 calcium channel blockers, 44 angiotensin-converting-enzyme inhibitors, 48 angiotensin II receptor blockers, and 40 used b-blockers). Sixty-two patients received insulin, whereas the rest 56 received per os antidiabetic medication (48 sulfonylureas, 34 biguanides, 19 glitazones, 6 dipeptidyl peptidase-4 inhibitors, and 6 glinides). Our study protocol was in conformity with the Helsinki Declaration of Human Rights and was approved by the Ethics Committee of the Scientific Council of the Medical School of the University of Alexandroupolis, Greece. All participants gave their written, informed consent.

### 2.2. Follow-Up and Endpoints

After enrolment, patients were followed over a period of seven years or the occurrence of death. Secondary outcome of the study was the occurrence of CV events. We obtained follow-up information for our study cohort from regular follow-up visits, hospital medical records, **and** death certificates and through an integrated telephone interview.

### 2.3. cIMT Measurement

cIMT was assessed at baseline, by a single, well-trained physician, using real-time B-mode ultrasonography, as described before [16].

### 2.4. Laboratory Analyses

To obtain whole blood, serum, and plasma, we collected blood samples from all participants, after an overnight fasting of 8 hours. Total, LDL, and high-density lipoprotein (HDL) cholesterol, triglycerides, creatinine, c-reactive protein (CRP), and glycated hemoglobin (HBA1c) were measured, as described elsewhere [17]. To assess ox-LDL, samples were centrifuged immediately, and plasma was stored at -20°C, until analysis. The concentration of ox-LDL in plasma was quantitated by the enzyme-linked immunosorbent assay (ELISA) method, following the manufacturer's instructions (human ox-LDL ELISA kit, Mercodia, Sweden), as was previously described [17]. Detection limit for ox-LDL assay was 0.3 U/L and intra/interassay coefficients of variation were <10%, according to the manufacturer. Albuminuria was evaluated as the urine albumin to creatinine ratio (UACR) in a morning, sterile, spot urine sample, as described before [18].

### 2.5. Genotyping of rs2741335 and rs11780592 *EPHX2* Polymorphisms

DNA extraction and genotyping have been extensively described elsewhere [19–21]. We performed a GTEX analysis (Supplementary Table 1) showing that both SNPs (rs11780592 and rs2741335) are associated with the expression of the EPHX2 gene in various tissues. Moreover, it appears that the top hit SNP for this gene is rs11780592, since it is associated with the gene's expression in most tissues, including sites of endothelial dysfunction development, such as arteries, aorta, heart, ventricles, brain, adipose tissue, and circulation. Therefore, the results of the GTEX analysis indicate that by studying this top hit SNP, we can hypothesize that we are studying the expression of the EPHX2 gene.

### 2.6. Statistical Analysis

We used the Kolmogorov-Smirnov test to test our data for normality. Binary variables are expressed as percent frequency, nonnormally distributed variables are expressed as median with interquartile range, and normally distributed variables as mean ± SD. Patients' characteristics were compared among different genotypes of rs11780592 *EPHX2* using chi-square for categorical variables, Mann–Whitney for nonnormally distributed variables, and independent *t*-test for normally distributed variables, as appropriate. Comparison of ox-LDL plasma levels among stages of diabetic CKD was performed with the Mann–Whitney test. To evaluate overall survival and CV events (fatal and nonfatal) for the rs2741335 and rs11780592 *EPHX2* genotypes, we used the Kaplan-Meier method and log-rank tests to compare survival curves. For survival analyses, all patients were categorized in two groups, according to rs11780592 *EPHX2* polymorphism: AA versus grouped AG and GG genotypes. We used univariate and multivariate Cox proportional hazard models (enter selection) to calculate adjusted hazard ratios (HRs) and 95% confidence intervals (CIs) for the associations between rs11780592 and rs2741335 *EPHX2* polymorphism and all-cause mortality and CV events. Models for all-cause mortality and CV events were adjusted for age, sex, and previous history of CV disease. Statistical analyses were performed by the IBM Statistical Package for Social Sciences (SPSS) 18.0, for Windows, Chicago, Illinois, USA. Significance was set at *p* < 0.05.

## 3. Results

As shown in Supplementary Table 2 (submitted in supplementary materials), the allelic and genotypic frequencies found in our cohort of Greeks are similar to those of Tuscan Italians from Southern Europe, which are a good reference for the Greek population, as described before [22]. Regarding other non-European populations, it is interesting to mention that East Asians do not have this variant, whereas Ashkenazi Jewish are the population with the greatest allele frequency for the G allele (minor allele frequency = 18%, gnomAD data). The Mann–Whitney test showed that ox-LDL levels were significantly different among CKD stages (*p* = 0.038). Baseline clinical, anthropometric, and biochemical characteristics of diabetic CKD patients according to rs11780592 *EPHX2* genotypes are shown in Table 1 (AA, AG, and GG genotypes) and Table 2 (AA versus grouped AG/GG). Compared to AG and GG, AA homozygotes had significantly increased diastolic and mean blood pressure levels (*p* = 0.007 and 0.038, respectively). This association was even more pronounced when AG and GG genotypes were grouped (Table 2). The grouped genotypes of rs11780592 EPHX2 polymorphism differed significantly also, among stages of diabetic CKD -G1/G2 as mild CKD, G3a/G3b as moderate CKD, G4 as severe CKD, and G4 as ESRD (*p* = 0.02), Supplementary Table 3. Age, body mass index (BMI), systolic blood pressure, glycated hemoglobin, and CRP did not differ significantly among groups. Compared to the other genotypes, there was an excess of male participants in the AA group (*p* = 0.04). Although lipid profile parameters (including total, LDL, and HDL cholesterol and triglycerides) were not significantly different among genotypes, compared to AG and GG, AA homozygotes presented significantly increased levels of plasma ox-LDL (*p* = 0.003, Table 1). UACR and cIMT were significantly different between the rs11780592 EPHX2 genotypes. Compared to AG and GG, AA homozygotes had higher values of both UACR and cIMT (*p* = 0.046 and *p* = 0.038, respectively). ox-LDL differed significantly among rs11780592 genotypes in G1/G2 and G4 stages of diabetic CKD, whereas cIMT was significantly higher in AA homozygotes (compared to AG and GG) in stages G4 and G5 (Supplementary Table 4). All the baseline characteristics of the patients did not differ significantly among rs2741335 *EPHX2* genotypes (results not shown).

**Figure 1:**
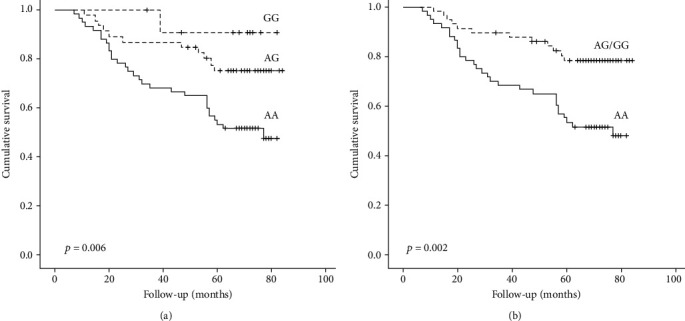
Kaplan-Meier curves for all-cause mortality according to rs11780592 EPHX2 polymorphism. (a) Comparison among patients with AA, AG, and GG genotypes. (b) Comparison of patients according to AA versus grouped AG/GG genotypes. Log-rank test, *p* = 0.006 and 0.002, respectively.

After the follow-up period, 42/118 patients died (30/60 with AA genotype, 11/42 with AG genotype, and 1/12 with GG genotype) and 49/118 experienced a new CV event (fatal or nonfatal). The Kaplan-Meier analysis revealed that patients with the AA genotype exhibited a significantly higher mortality risk, compared to patients with AG and GG genotypes (Figure 1(a), *p* = 0.006). This association became even stronger, when AG and GG genotypes were grouped (Figure 1(b), AA vs. AG/GG, *p* = 0.002). AA homozygotes were strongly associated with all-cause mortality in both univariate (HR = 2.74, CI = 1.40–5.35, *p* = 0.003, Table 3) and multivariate Cox regression analysis (HR = 2.61, CI = 1.32–5.17, *p* = 0.006, Table 3). However, both the Kaplan-Meier and Cox regression analysis failed to show any association between rs11780592 *EPHX2* polymorphism and the occurrence of CV events (Table 3). Moreover, rs2741335 *EPHX2* polymorphism was not associated with any of the study outcomes.

## 4. Discussion

Diabetic CKD is characterized by increased mortality and CV disease rates. This is partially attributed to the enhanced OS and inflammation that is triggered by diabetes and CKD. Oxidation of LDL cholesterol is the first crucial step towards endothelial dysfunction, the hallmark of atherosclerosis. Thus, it is hypothesized that ox-LDL might be not only a marker of OS but also a biomarker reflecting arterial health. Having these in mind, the identification of genetic biomarkers that might determine circulating ox-LDL levels and predict mortality might be of utmost clinical importance. Arachidonic acid-derived epoxyeicosatrienoic acids are anti-inflammatory, antioxidant agents with established beneficial effects on CV disease. Hydration of these molecules by soluble EPHX2 is the main pathway of their metabolic degradation to the less active, diol molecules. By catalyzing this reaction, soluble EPHX2 promotes inflammation, OS, and atherosclerosis. The EPHX2 gene is a protein-coding gene with genomic location in the short arm of chromosome 8 (8p21.2-p21.1). Genetic variance of the gene encoding EPHX2 has been repeatedly associated with CV outcomes; however, until to date, the data regarding the association between *EPHX2* gene polymorphisms, ox-LDL, and mortality remain extremely limited.

In a cohort of 118 patients with established diabetic CKD, we found a strong association between cIMT and rs11780592 *EPHX2* polymorphism. cIMT is a well-established surrogate marker for endothelial dysfunction and subclinical atherosclerosis in CKD patients and has been associated with mortality and CV disease in a population similar to ours [16] and patients undergoing maintenance HD [23]. In agreement with our results, in vivo data from animal and human cultured carotid artery smooth muscle cells suggest that soluble EPHX2 might promote atherosclerotic progression and arterial remodelling after acute injury [9]. The authors hypothesized that since EPHX2 is involved in the phenotypic transformation of vascular smooth muscle cells that leads to endothelial dysfunction and atherosclerosis, inhibition of its activity might be a potential therapeutic target for ameliorating carotid atherosclerosis and CV disease. Likewise, in T2DM patients, Duflot et al. showed that increased metabolic breakdown of epoxyeicosatrienoic acids by EPHX2 and impaired bioavailability of nitric oxide are tightly associated with impaired conduit artery endothelial function, independently of their blood pressure status [24]. Moreover, these authors found that among hypertensive patients, only diabetics but not nondiabetics presented a significant elevation of circulating reactive oxygen species (a marker of OS) and decreased nitric oxide release. Furthermore, inhibition of EPHX2 might have pleiotropic beneficial effects on hypertension, endothelial dysfunction, OS, and CV disease. Recently, three novel agents acting as EPHX2 inhibitors were identified as potent vasodilators [25], whereas clinical data suggest that genetic variation in EPHX2 might be associated with forearm vasodilation, a marker of endothelial function [26]. Thus, in agreement with our study, there is a growing body of evidence suggesting a central role for EPHX2 in the regulation of endothelial function and the pathogenesis of atherosclerosis. In our study, the AA genotype of rs11780592 was associated with increased thickness of the carotid wall, when compared to AG and GG genotypes. The association remained significant after grouping for the G allele. Although several studies have examined other *EPHX2* polymorphisms, there are no existing data in the literature regarding rs11780592 and rs2741335 polymorphisms.

Another finding of our study was the association between rs11780592 *EPHX2* polymorphism and albuminuria. Silencing of the *EPHX2* gene in mice has been shown to attenuate renal inflammation and albuminuria [27], whereas experimental and clinical data have shown that epoxyeicosatrienoic acids and genetic variations of the *EPHX2* gene were directly associated with insulin sensitivity and glucose homeostasis [28]. Moreover, in diabetic, overweight animal models, inhibition of EPHX2 prevents albuminuria, independently from its glucose-lowering effect [29]. Diabetic CKD is characterized by a gradual reduction of eGFR and presence of albuminuria. In a case-control study, Ma et al. found that the coding rs751141 *EPHX2* polymorphism is associated with diabetic CKD in a cohort of Chinese T2DM population [30]. The association between genetic variation of the *EPHX2* gene and kidney outcomes has been also highlighted by two separate studies, in renal transplant recipients [31, 32]. These studies suggested that donor genetic variability in the *EPHX2* gene might be predictive of graft dysfunction and acute rejection in kidney transplant recipients. In our study, we also found that plasma ox-LDL levels were associated with the stage of diabetic CKD, as defined by albuminuria and eGFR. It is known that OS is present even at the early stages of CKD and progresses along with disease progression to ESRD. In agreement with our findings, Dounousi et al. reported that circulating 8-isoprostanes, a marker of lipid peroxidation status, increased significantly as CKD progressed and were strongly correlated with the degree of renal function [33].

The main finding of our study was that rs11780592 *EPHX2* polymorphism was associated with ox-LDL, cIMT, and mortality, in a cohort of patients with diabetic CKD. Compared to AG and GG, AA homozygotes presented significantly increased plasma ox-LDL levels, increased cIMT values, and increased mortality rates. We hypothesize that the association between *EPHX2* gene polymorphism and mortality might be attributed to the increased circulating ox-LDL levels. In agreement with our findings, in apolipoprotein E knockout mice, inhibition of EPHX2 significantly decreased the onset and development of atherosclerotic lesions. These antiatherogenic, beneficial effects of EPHX2 inhibitors were attributed to a significant decrease in LDL and elevation of HDL cholesterol [34]. Moreover, blocking EPHX2 was accompanied by a significant improvement of hypertension and endothelial function. Therefore, by reducing LDL cholesterol and OS status, EPHX2 inhibitors might decrease ox-LDL formation. In animal models with ischemic stroke, administration of selective EPHX2 inhibitors was found to improve clinical outcomes, by suppressing OS and inflammation [35]. The tight association between EPHX2 and ox-LDL was also highlighted in another animal study, where blocking of soluble EPHX2 was accompanied by a rise in *β*-oxidation of fatty acids [36]. In vitro, incubation of foam cells with a novel inhibitor of soluble EPHX2 caused a significant decrease in cholesterol accumulation in ox-LDL-loaded macrophages [37], possibly by increasing the circulating epoxyeicosatrienoic fatty acids. Inhibition of soluble EPHX2 promoted cholesterol efflux and prevented the internalization of ox-LDL cholesterol.

There is a growing body of evidence suggesting a direct association between soluble EPHX2 and CV disease. This association might be attributed to the increased degradation of the cardioprotective epoxyeicosatrienoic acids, to the promotion of OS and inflammation, and to the induction of endothelial dysfunction. Large epidemiologic studies (the Atherosclerosis Risk in Communities and the Diabetes Heart Study) reported that rs7837347, rs7003694, rs747276, and rs41507953 *EPHX2* gene polymorphisms were associated with subclinical CV disease and coronary heart disease [10, 11]. However, in our study, although only rs11780592 *EPHX2* polymorphism was associated with mortality, no association was found between the two polymorphisms and CV disease.

To the best of our knowledge, this is the first study evaluating the association of rs11780592 and rs2741335 *EPHX2* gene polymorphisms with ox-LDL, carotid atherosclerosis, and mortality in a cohort of patients with diabetic CKD. However, our study has certain limitations. First, the cross-sectional design of the study precludes establishing causality. Second, the relatively small sample size and the lack of data on soluble EPHX2 levels and activity are also recognized as limitations. Since there are no data regarding these two polymorphisms in the literature, further, larger epidemiologic studies are needed in order to fully elucidate the role of *EPHX2* gene variations. Identification of genetic markers might provide a deeper understanding of the molecular pathways involved in OS and atherosclerosis in diabetic CKD, which subsequently might lead to the recognition of novel therapeutic targets.

## 5. Conclusions

In conclusion, our study demonstrated that in a cohort of 118 T2DM patients with various degrees of CKD, including ESRD, rs11780592 *EPHX2* polymorphism was associated with increased circulating ox-LDL, increased cIMT, and all-cause mortality. The AA genotype of rs11780592 polymorphism was associated with OS, carotid atherosclerosis, and all-cause mortality in these patients. Since EPHX2 regulates the cholesterol efflux and the oxidation of LDL in foam cells and macrophages, our study suggests that a genetic basis to endothelial dysfunction and OS might be present in diabetic CKD.

## Figures and Tables

**Table 1:** Association of rs11780592 EPHX2 polymorphism with anthropometric, clinical, and biochemical characteristics of patients with diabetic chronic kidney disease.

	rs11780592 EPHX2 genotypes			*p*
*N* = 118	AA (*n* = 60)	AG (*n* = 46)	GG (*n* = 12)	
Age (years)	68.6 (8.1)	67.3 (9.4)	65.8 (8.8)	0.47
Gender (M/F)	34/26	17/29	3/9	**0.04**
BMI (kg/m^2^)	31.4 (5.1)	31.1 (5.7)	29.7 (4.2)	0.45
SBP (mmHg)	140.9 (18.9)	136.2 (20.8)	135.4 (22.7)	0.35
DBP (mmHg)	79.5 (9.2)	75.3 (11.0)	71.4 (10.5)	**0.007**
Mean BP (mmHg)	99.9 (11.1)	95.6 (12.9)	92.7 (12.8)	**0.038**
Duration of T2DM (years)	16.3 (7.8)	13.1 (7.2)	14.8 (8.3)	0.10
HbA1c (%)	7.5 (1.2)	7.3 (0.9)	7.7 (1.3)	0.59
Total cholesterol (mg/dL)	183.7 (47.9)	168.2 (45.6)	159.6 (30.1)	0.15
LDL cholesterol (mg/dL)	104.0 (41.3)	93.1 (35.9)	81.6 (22.0)	0.22
HDL cholesterol (mg/dL)	44.8 (11.9)	44.5 (11.9)	52.1 (19.3)	0.45
Triglycerides (mg/dL)	157.5 (66-450)	141.5 (52-551)	93.0 (69-315)	0.12
History of CV disease (%)	70	74	58.3	0.57
CRP (mg/dL)	0.25 (0-11)	0.20 (0-4)	0.20 (0-4.5)	0.72
eGFR (mL/min/1.73 m^2^)	43.6 (30.0)	49.5 (34.9)	60.9 (34.2)	0.27
UACR (mg/g)	66 (3-7000)	35.5 (7-2600)	20.3 (2.4-250)	**0.046**
Mean cIMT (mm)	0.94 (0.55-1.76)	0.89 (0.55-1.78)	0.83 (0.56-0.95)	**0.031**
ox-LDL (U/L)	68.3 (17.9-123.4)	53.1 (22.0-83.4)	52.9 (22.9-92.2)	**0.003**

*p* values of Mann–Whitney, *t*-test, or chi-square test for differences of variables among rs11780592 EPHX2 genotypes. BMI: body mass index; SBP: systolic blood pressure; DBP: diastolic blood pressure; mean BP: mean blood pressure; T2DM: type 2 diabetes mellitus; HbA1c: glycated hemoglobin A1c; LDL: low-density lipoprotein; HDL: high-density lipoprotein; CV: cardiovascular; CRP: C-reactive protein; eGFR: estimated glomerular filtration rate; UACR: urinary albumin-creatinine ratio; cIMT: carotid intima-media thickness; ox-LDL: oxidized low-density lipoprotein.

**Table 2:** Association of rs11780592 EPHX2 grouped genotypes with anthropometric, clinical, and biochemical characteristics of patients with diabetic chronic kidney disease.

	rs11780592 EPHX2 genotypes, grouped		*p*
*N* = 118	AA (*n* = 60)	AG & GG (*n* = 58)	
Age (years)	68.6 (8.1)	67.0 (9.2)	0.31
Gender (M/F)	34/26	20/38	**0.013**
BMI (kg/m^2^)	31.4 (5.1)	30.8 (5.5)	0.34
SBP (mmHg)	140.9 (18.9)	136.1 (21.0)	0.15
DBP (mmHg)	79.5 (9.2)	74.5 (10.9)	**0.003**
Mean BP (mmHg)	99.9 (11.1)	95 (12.8)	**0.013**
Duration of T2DM (years)	16.3 (7.8)	13.5 (7.3)	**0.04**
HbA1c (%)	7.5 (1.2)	7.4 (1.0)	0.52
Total cholesterol (mg/dL)	183.7 (47.9)	166.4 (42.7)	0.06
LDL cholesterol (mg/dL)	104.0 (41.3)	90.7 (33.7)	0.14
HDL cholesterol (mg/dL)	44.8 (11.9)	46.1 (13.9)	0.52
Triglycerides (mg/dL)	157.5 (66-450)	137 (52-551)	**0.07**
History of CV disease (%)	70	70.6	0.55
CRP (mg/dL)	0.25 (0-11)	0.20 (0-4.5)	0.49
eGFR (mL/min/1.73 m^2^)	43.6 (30.0)	51.9 (34.7)	0.22
UACR (mg/g)	66 (3-7000)	27.5 (2.4-2600)	0.055
Mean cIMT (mm)	0.94 (0.55-1.76)	0.86 (0.55-1.78)	**0.038**
ox-LDL (U/L)	68.3 (17.9-123.4)	53.1 (22-92.2)	**0.001**

*p* values of Mann–Whitney, *t*-test, or chi-square test for differences of variables among rs11780592 EPHX2 genotypes. BMI: body mass index; SBP: systolic blood pressure; DBP: diastolic blood pressure; mean BP: mean blood pressure; T2DM: type 2 diabetes mellitus; HbA1c: glycated hemoglobin A1c; LDL: low-density lipoprotein; HDL: high-density lipoprotein; CV: cardiovascular; CRP: C-reactive protein; eGFR: estimated glomerular filtration rate; UACR: urinary albumin-creatinine ratio; cIMT: carotid intima-media thickness; ox-LDL: oxidized low-density lipoprotein.

**Table 3:** Cox proportional hazard analysis (enter regression) showing predictors for all-cause mortality in univariate and multivariate models, in patients with rs11780592 EPHX2 and rs2741335 EPHX2 polymorphism.

		HR	CI	*p*
*All-cause mortality*				
Model 1^a^				
	rs11780592 EPHX2	2.74	1.40-5.35	**0.003**
	rs2741335 EPHX2	0.85	0.58-1.26	0.42
Model 2^b^				
	rs11780592 EPHX2	2.61	1.32-5.17	**0.006**
*CV events (fatal and nonfatal)*				
Model 1^a^				
	rs11780592 EPHX2	1.10	0.72-1.69	0.67
	rs2741335 EPHX2	0.77	0.54-1.11	0.16

Model 1^a^ = univariate model. Model 2^b^ = multivariate model, adjusted for age, sex, and previous history of cardiovascular disease. HR = hazard ratio; CI 95% = confidence interval.

## Data Availability

The data used to support the findings of this study are included within the article.

## Supplementary Materials

Supplementary Materials Supplementary Materials include 4 tables. Supplementary Table 1: analysis of the associated variants on tissue expression. Supplementary Table 2: allelic and genotypic frequencies in the European populations of the 1000 Genomes dataset. Supplementary Table 3: distribution of rs11780592 EPHX2 polymorphism genotypes in different stages of diabetic CKD. Supplementary Table 4: association of rs11780592 EPHX2 polymorphism with ox-LDL and cIMT among different stages of diabetic CKD.
