# Supplementary material for: Association of rs11780592 Polymorphism in the Human Soluble Epoxide Hydrolase Gene (EPHX2) with Oxidized LDL and Mortality in Patients with Diabetic Chronic Kidney Disease
Source: Oxid Med Cell Longev. 2021 May 6;2021:8817502. doi: 10.1155/2021/8817502 (PMC8121583; doi:10.1155/2021/8817502)
Supplement: Supplementary Materials — include 4 tables. Supplementary Table 1: analysis of the associated variants on tissue expression. Supplementary Table 2: allelic and genotypic frequencies in the European populations of the 1000 Genomes dataset. Supplementary Table 3: distribution of rs11780592 EPHX2 polymorphism genotypes in different stages of diabetic CKD. Supplementary Table 4: association of rs11780592 EPHX2 polymorphism with ox-LDL and cIMT among different stages of diabetic CKD. [file 8817502.f1.docx]

**Supplementary Table 1.** Analysis of the associated variants on tissue expression. We report the significant associations between SNP genotypes and gene expression level on the reported tissue

| **Gencode Id** | **Gene Symbol** | **Variant Id** | **SNP Id** | **P-value** | **NES** | **Tissue** |
| --- | --- | --- | --- | --- | --- | --- |
| ENSG00000120915.13 | EPHX2 | chr8_27561230_A_G_b38 | rs11780592 | 2,1E-16 | 0,28 | Artery - Tibial |
| ENSG00000120915.13 | EPHX2 | chr8_27561230_A_G_b38 | rs11780592 | 8,9E-12 | 0,23 | Esophagus - Muscularis |
| ENSG00000120915.13 | EPHX2 | chr8_27561230_A_G_b38 | rs11780592 | 8,9E-12 | 0,24 | Lung |
| ENSG00000120915.13 | EPHX2 | chr8_27561230_A_G_b38 | rs11780592 | 1,1E-11 | 0,27 | Cells - Cultured fibroblasts |
| ENSG00000120915.13 | EPHX2 | chr8_27561230_A_G_b38 | rs11780592 | 3,8E-10 | 0,21 | Skin - Sun Exposed (Lower leg) |
| ENSG00000120915.13 | EPHX2 | chr8_27561230_A_G_b38 | rs11780592 | 1,4E-09 | 0,25 | Esophagus - Gastroesophageal Junction |
| ENSG00000120915.13 | EPHX2 | chr8_27561230_A_G_b38 | rs11780592 | 4,8E-09 | 0,18 | Thyroid |
| ENSG00000120915.13 | EPHX2 | chr8_27561230_A_G_b38 | rs11780592 | 0,000000011 | 0,24 | Skin - Not Sun Exposed (Suprapubic) |
| ENSG00000120915.13 | EPHX2 | chr8_27561230_A_G_b38 | rs11780592 | 0,000000015 | 0,24 | Muscle - Skeletal |
| ENSG00000120903.12 | CHRNA2 | chr8_27561230_A_G_b38 | rs11780592 | 0,000000022 | 0,67 | Brain - Cerebellum |
| ENSG00000120915.13 | EPHX2 | chr8_27561230_A_G_b38 | rs11780592 | 0,000000023 | 0,19 | Nerve - Tibial |
| ENSG00000234770.1 | GULOP | chr8_27561230_A_G_b38 | rs11780592 | 0,000000043 | -0,34 | Skin - Not Sun Exposed (Suprapubic) |
| ENSG00000234770.1 | GULOP | chr8_27561230_A_G_b38 | rs11780592 | 0,000000093 | -0,32 | Skin - Sun Exposed (Lower leg) |
| ENSG00000120915.13 | EPHX2 | chr8_27561230_A_G_b38 | rs11780592 | 0,0000001 | 0,19 | Adipose - Visceral (Omentum) |
| ENSG00000120915.13 | EPHX2 | chr8_27561230_A_G_b38 | rs11780592 | 0,00000016 | 0,24 | Artery - Aorta |
| ENSG00000120915.13 | EPHX2 | chr8_27561230_A_G_b38 | rs11780592 | 0,00000021 | 0,38 | Spleen |
| ENSG00000120915.13 | EPHX2 | chr8_27561230_A_G_b38 | rs11780592 | 0,00000029 | 0,25 | Heart - Atrial Appendage |
| ENSG00000120903.12 | CHRNA2 | chr8_27561230_A_G_b38 | rs11780592 | 0,0000011 | 0,61 | Brain - Cerebellar Hemisphere |
| ENSG00000120915.13 | EPHX2 | chr8_27561230_A_G_b38 | rs11780592 | 0,0000035 | 0,21 | Esophagus - Mucosa |
| ENSG00000120915.13 | EPHX2 | chr8_27561230_A_G_b38 | rs11780592 | 0,0000045 | 0,31 | Pituitary |
| ENSG00000120915.13 | EPHX2 | chr8_27561230_A_G_b38 | rs11780592 | 0,000015 | 0,12 | Colon - Transverse |
| ENSG00000234770.1 | GULOP | chr8_27561230_A_G_b38 | rs11780592 | 0,000019 | -0,42 | Small Intestine - Terminal Ileum |
| ENSG00000120915.13 | EPHX2 | chr8_27561230_A_G_b38 | rs11780592 | 0,000024 | 0,18 | Heart - Left Ventricle |
| ENSG00000147419.17 | CCDC25 | chr8_27561230_A_G_b38 | rs11780592 | 0,000026 | 0,26 | Brain - Nucleus accumbens (basal ganglia) |
| ENSG00000120915.13 | EPHX2 | chr8_27561230_A_G_b38 | rs11780592 | 0,00006 | 0,15 | Colon - Sigmoid |
| ENSG00000120915.13 | EPHX2 | chr8_27561230_A_G_b38 | rs11780592 | 0,000071 | 0,2 | Testis |
| ENSG00000120915.13 | EPHX2 | chr8_27561230_A_G_b38 | rs11780592 | 0,000083 | 0,11 | Whole Blood |
| ENSG00000120903.12 | CHRNA2 | chr8_27561230_A_G_b38 | rs11780592 | 0,00011 | 0,15 | Whole Blood |
| ENSG00000120903.12 | CHRNA2 | chr8_27480274_A_C_b38 | rs2741335 | 4,4E-14 | 0,73 | Brain - Cerebellum |
| ENSG00000120903.12 | CHRNA2 | chr8_27480274_A_C_b38 | rs2741335 | 5,8E-09 | 0,61 | Brain - Cerebellar Hemisphere |
| ENSG00000120899.17 | PTK2B | chr8_27480274_A_C_b38 | rs2741335 | 0,0000034 | 0,15 | Artery - Tibial |
| ENSG00000120915.13 | EPHX2 | chr8_27480274_A_C_b38 | rs2741335 | 0,000022 | 0,39 | Brain - Cerebellar Hemisphere |
| ENSG00000120915.13 | EPHX2 | chr8_27480274_A_C_b38 | rs2741335 | 0,000041 | 0,15 | Muscle - Skeletal |
| ENSG00000120899.17 | PTK2B | chr8_27480274_A_C_b38 | rs2741335 | 0,000041 | 0,13 | Artery - Aorta |
| ENSG00000168079.16 | SCARA5 | chr8_27480274_A_C_b38 | rs2741335 | 0,000058 | -0,11 | Adipose - Subcutaneous |
| ENSG00000120903.12 | CHRNA2 | chr8_27480274_A_C_b38 | rs2741335 | 0,000072 | 0,14 | Whole Blood |

**Supplementary Table 2.** Allelic and genotypic frequencies in the European populations of the 1000 Genomes dataset.

| **Population** | **SNP** | **Allele Frequency** | **Genotype Frequency** |
| --- | --- | --- | --- |
| EUR | rs11780592 | A: 0.829 (834) G: 0.171 (172) | A\|A: 0.686 (345) A\|G: 0.286 (144) G\|G: 0.028 (14) |
| CEU | rs11780592 | A: 0.864 (171) G: 0.136 (27) | A\|A: 0.737 (73) A\|G: 0.253 (25) G\|G: 0.010 (1) |
| FIN | rs11780592 | A: 0.859 (170) G: 0.141 (28) | A\|A: 0.737 (73) A\|G: 0.242 (24) G\|G: 0.020 (2) |
| GBR | rs11780592 | A: 0.841 (153) G: 0.159 (29) | A\|A: 0.703 (64) A\|G: 0.275 (25) G\|G: 0.022 (2) |
| IBS | rs11780592 | A: 0.832 (178) G: 0.168 (36) | A\|A: 0.692 (74) A\|G: 0.280 (30) G\|G: 0.028 (3) |
| TSI | rs11780592 | A: 0.757 (162) G: 0.243 (52) | A\|A: 0.570 (61) A\|G: 0.374 (40) G\|G: 0.056 (6) |
|  |  |  |  |
| **Population** | **SNP** | **Allele Frequency** | **Genotype Frequency** |
| EUR | rs2741335 | A: 0.714 (718) C: 0.286 (288) | A\|A: 0.509 (256) A\|C: 0.410 (206) C\|C: 0.082 (41) |
| CEU | rs2741335 | A: 0.727 (144) C: 0.273 (54) | A\|A: 0.485 (48) A\|C: 0.485 (48) C\|C: 0.030 (3) |
| FIN | rs2741335 | A: 0.692 (137) C: 0.308 (61) | A\|A: 0.465 (46) A\|C: 0.455 (45) C\|C: 0.081 (8) |
| GBR | rs2741335 | A: 0.709 (129) C: 0.291 (53) | A\|A: 0.527 (48) A\|C: 0.363 (33) C\|C: 0.110 (10) |
| IBS | rs2741335 | A: 0.729 (156) C: 0.271 (58) | A\|A: 0.551 (59) A\|C: 0.355 (38) C\|C: 0.093 (10) |
| TSI | rs2741335 | A: 0.710 (152) C: 0.290 (62) | A\|A: 0.514 (55) A\|C: 0.393 (42) C\|C: 0.093 (10) |

EUR: All Europeans, CEU: Northwestern Europeans, FIN: Finnish, GBR: British, IBS: Iberians, TSI: Tuscan Italians from Southern Europe

**Supplementary Table 3**. The distribution of rs11780592 *EPHX2* polymorphism genotypes in different stages of diabetic CKD -G1/G2 as mild CKD, G3a/G3b as moderate CKD, G4 as severe CKD and G5 as ESRD.

|  | ***rs11780592 EPHX2 genotypes*** | | | ***P*** | ***rs11780592 genotypes grouped*** | | ***P*** |
| --- | --- | --- | --- | --- | --- | --- | --- |
| ***Stages of diabetic CKD*** | **AA**  **(n=60)** | **AG**  **(n=46)** | **GG**  **(n=12)** | 0.07 | **AA**  **(n=60)** | **AG/GG**  **(n=58)** | **0.02** |
| G1/G2 (n=45) | 17 | 21 | 7 |  | 17 | 28 |  |
| G3a/G3b (n=33) | 21 | 10 | 2 |  | 21 | 12 |  |
| G4 (n=11) | 9 | 1 | 1 |  | 9 | 2 |  |
| G5 (n=29) | 13 | 14 | 2 |  | 13 | 16 |  |

**Supplementary Table 4**. The association of rs11780592 *EPHX2* polymorphism with ox-LDL and cIMT among different stages of diabetic CKD.

| **rs11780592 EPHX2 polymorphism in different *Stages of diabetic CKD*** | ***Ox-LDL*** | ***cIMT*** |
| --- | --- | --- |
| rs11780592 EPHX2 polymorphism in G1/G2 (n=45) | **P=0.016** | P=0.50 |
| rs11780592 EPHX2 polymorphism in G3a/G3b (n=33) | P=0.26 | P=0.27 |
| rs11780592 EPHX2 polymorphism in G4 (n=11) | **P=0.02** | **P=0.05** |
| rs11780592 EPHX2 polymorphism in G5 (n=29) | P=0.74 | **P=0.04** |
